# Influenza infection and risk of acute pulmonary embolism

**DOI:** 10.1186/1477-9560-5-16

**Published:** 2007-10-16

**Authors:** Matthijs van Wissen, Tymen T Keller, Brechje Ronkes, Victor EA Gerdes, Hans L Zaaijer, Eric CM van Gorp, Dees PM Brandjes, Marcel Levi, Harry R Büller

**Affiliations:** 1 Department of Vascular Medicine, Academic Medical Center, Amsterdam, The Netherlands; 2 Department of Internal Medicine, Slotervaart Hospital, Amsterdam, The Netherlands; 3 Department of Microbiology, Academic Medical Center, Amsterdam, The Netherlands

## Abstract

**Background:**

Influenza infections have been associated with procoagulant changes. Whether influenza infections lead to an increased risk of pulmonary embolism remains to be established.

**Methods:**

We conducted a nested case control study in a large cohort of patients with a clinical suspicion of having pulmonary embolism. Blood samples were collected to investigate the presence of influenza A and B by complement fixation assay (CFA). We compared case patients, in whom pulmonary embolism was proven (n = 102), to controls, in whom pulmonary embolism was excluded (n = 395). Furthermore, we compared symptoms of influenza-like illness in both patient groups 2 weeks prior to inclusion in the study, using the influenza-like illness (ILI) score, which is based on a questionnaire. We calculated the risk of pulmonary embolism associated with influenza infection.

**Results:**

The percentage of patients with influenza A was higher in the control group compared to the case group (4.3% versus 1.0%, respectively, odds ratio 0.22; 95% CI: 0.03–1.72). Influenza B was not detectable in any of the cases and was found in 3 of the 395 controls (0.8%). The ILI score was positive in 24% of the cases and 25% in the control persons (odds ratio 1.16, 95% CI: 0.67–2.01). We did not observe an association between the ILI score and proven influenza infection.

**Conclusion:**

In this clinical study, influenza infection was not associated with an increased risk of acute pulmonary embolism. The ILI score is non-specific in this clinical setting.

## Background

Deep vein thrombosis and pulmonary embolism, collectively known as venous thromboembolism (VTE), have an annual incidence of approximately 2–3 per 1000 people [1]. Many risk factors of venous thromboembolism have been well established, including genetic predisposition, immobilization, surgery, pregnancy, oral contraceptives and malignancies. However, there are patients who develop venous thromboembolism in the absence of one of these risk factors. There is growing evidence that acute infections are associated with an increased risk of developing a venous thromboembolic event.

Emmerich and others found an association between positive antibody titers for Chlamydia pneumoniae and venous thromboembolism [2-4]. Also, patients with HIV are at increased risk of developing venous thromboembolism [5]. In a population-based case-control study, subjects with elevated IL-8 levels had an increased risk of venous thrombosis, which indicates the role of inflammation (potentially as a consequence of infection) in the pathogenesis of venous thrombosis [6].

Recently, it has been observed that acute urinary tract infection and signs of respiratory tract infection are associated with an increased the risk of venous thromboembolism [7]. The risk was highest during the 2 weeks after a urinary tract infection, with an incidence ratio for deep vein thrombosis of 2.10 (95% CI 1.56–2.82), and that for PE of 2.11 (1.38–3.23), and gradually falling over the subsequent months. The incidence ratio for deep vein thrombosis in patients with signs of a respiratory tract infection was 1.91 (95% CI: 1.49–2.44). Possible diagnostic misclassification of an early presentation of PE as a respiratory infection precluded a reliable estimate of the risk of PE after respiratory tract infection.

It is known since Virchov that venous thromboembolism is usually the result of either a procoagulant state of the blood, or dysfunction of the vessel wall, eventually in combination with reduced flow of the blood. It has been shown in vitro that influenza affects each of these factors. Visseren et al. demonstrated that monocytes and endothelial cells that were incubated with influenza are able to activate coagulation, causing an average 55% reduction in clotting time and a 4- to 5-fold increase in the expression of tissue factor [8,9]. There are also indications that influenza leads to a procoagulant state of the blood in vivo. Recently, we have shown that influenza infections in elderly people result in increased levels of the prohemostatic proteins von Willebrand factor and plasmin-α2-antiplasmin (PAP) [10]. It is further known that inflammation leads to down regulation of thrombomodulin and the endothelial cell protein C receptor (EPCR) via different cytokines, thereby reducing the ability to generate the natural anticoagulant activated protein C (APC). Furthermore, inflammation induces inhibition of fibrinolysis by elevated levels of plasminogen activator inhibitor 1 (PAI-1), and via inflammatory mediators like interleukin 6 it increases the number of platelets and the newly formed thrombocytes appear to be more thrombogenic [11].

There are few data on the possible association between acute respiratory tract infections caused by influenza virus and the occurrence of venous thromboembolism. We hypothesized that influenza infections lead to a pro-coagulant state of the blood, resulting in a higher risk of pulmonary embolism. Therefore, we assessed the frequency of influenza infection among patients with a clinical suspicion of acute pulmonary embolism. Furthermore, we established whether the presence of signs and symptoms of an acute influenza-like illness (ILI) are specific in the clinical setting of patients suspect for having acute pulmonary embolism.

## Methods

### Study design

We performed a nested case control study in the cohort of the ANTELOPE study. The ANTELOPE study was performed from May 1999 to April 2001 in three hospitals in the Netherlands, as described in more detail previously [12]. In this study, in- and outpatients, with clinically suspected acute pulmonary embolism, were prospectively investigated. The diagnosis of pulmonary embolism was confirmed by a high probability perfusion-ventilation (V/Q) scan.

From this cohort we selected case patients, in whom pulmonary embolism was proven and we compared them to controls from the same cohort, in whom pulmonary embolism was excluded.

### Laboratory investigations

Blood samples were taken on the day of inclusion in the ANTELOPE study. Follow-up samples were taken 2–3 weeks later (convalescent phase). Influenza detection in serum samples was performed by a complement fixation assay (CFA). A recent influenza infection is suggested by a fourfold rise in complement fixation titre in paired samples or by a single high titre [13]. Therefore, a dilution of 1:64 or more was considered to be highly suggestive for recent influenza A and/or B infection and regarded as a positive test result.

### Influenza-like illness

At baseline we recorded symptoms of a respiratory tract infection, including: acute onset of fever (temperature ≥ 37,8°C), subjective feeling of fever or chills, cough with or without sputum, rhinitis, headache and sore throat. From these symptoms an influenza-like illness (ILI) score was calculated, based on the recommendations of previous studies on clinical signs and symptoms predicting influenza infection [14,15]. The ILI score was considered positive when there was an acute onset of fever (> 37.8°C) plus at least two of the above mentioned symptoms two weeks or less prior to inclusion.

### Statistical analysis

Results are presented as means plus or minus standard deviation (SD) and in percentages. Odds ratios (OR) and corresponding 95% confidence intervals (95% CI), to indicate the association between influenza infection and pulmonary embolism, were calculated using logistic regression analysis. Odds ratios were adjusted for age, chronic obstructive pulmonary disease (COPD) and asthma.

## Results

In this study a total of 497 patients were included, 102 patients with an acute pulmonary embolism and 395 patients in which a pulmonary embolism was excluded. Baseline characteristics of the pulmonary embolism group and non-pulmonary embolism group are shown in table 1.

**Table 1:** Baseline characteristics.

	Baseline characteristics	
	
	Case Group (n = 102)	Control Group (n = 395)
Sex:		
Male	46 (45.1%)	139 (35.2%)
Female	56 (54.9%)	216 (54.7%)
Age	58 (SD 17.1)	53 (SD17.6)
COPD	5 (4.9%)	42 (10.6%)
Asthma	5 (4.9%)	38 (9.6%)
Atherosclerotic disease	10 (9.8%)	47 (11.9%)
Malignancy	23 (22.5%)	54 (13.7%)
Immobilisation	16 (15.7%)	42 (10.6%)
Recent surgery	20 (19.0%)	56 (14.2%)
Recent trauma	2 (2.0%)	4 (1.0%)
Oral anticonceptives	12 (11.8%)	47 (11.9%)
History of VTE	19 (18.6%)	35 (8.9%)
Family history of CVD	15 (14.7%)	40 (10.1%)
Smoking	11 (10.8%)	74 (18.7%)

Baseline characteristics of pulmonary embolism group versus non-pulmonary embolism group: frequencies with Standard Deviation (SD) and percentages between brackets. Immobilisation: complete immobilisation for at least 3 days during the 4 weeks prior to inclusion. COPD: Chronic Obstructive Pulmonary Disease. CVD = Cardio Vascular Disease. VTE = venous thromboembolism

### Number of influenza infections

The percentage of patients with a positive result of the CFA for influenza A was higher in the non-pulmonary embolism group (17/395 (4.3%)) compared to the pulmonary embolism group (1/102 (1.0%)). The odds ratio was 0.22; 95% CI: 0.03–1.72. Influenza B was not detectable in any of the pulmonary embolism patients and in 3 of the 395 non-pulmonary embolism patients (0.8%). We did not calculate the odds ratio for influenza B, because of this very low number of patients with influenza B.

Vaccination has not been recorded in this study. In the Netherlands, patients are considered for vaccination against influenza from the age of 65 (on a voluntary basis), or when they belong to certain risk groups, such as patients with COPD or cardiovascular disease. From the pulmonary embolism group, 39 subjects (38%) were older than 65 years versus 103 subjects (26%) in the control group. The frequency of subjects that belong to such a risk group was higher in the control group (COPD 4,9% versus 10.6% and atherosclerotic disease 9.8% versus 11.9% in the cases and controls respectively).

### Symptoms of respiratory tract infection

The influenza-like illness score was positive in 24% of the pulmonary embolism patients (24/102). In the non-pulmonary embolism group this was the case for 25% (99/395). The adjusted odds ratio for the influenza-like illness score was 1.16; 95% CI: 0.67–2.01. We did not observe a clear association between the influenza-like illness score and proven influenza detection by CFA in either group.

## Discussion

In this case control study we assessed the frequency of influenza in a cohort of patients with a clinical suspicion of pulmonary embolism and demonstrate that influenza A infection is rare among patients with a proven pulmonary embolism, while it does occur significantly more often in the group of patients without pulmonary embolism. Symptoms of influenza, detected by the influenza-like illness score, were frequently present in both patient groups. Therefore, we could not establish an association between influenza, which causes respiratory tract infections, and an increased risk of pulmonary embolism. Our results indicate that influenza is not an important preceding factor for pulmonary embolism and that symptoms of influenza are non-specific in this clinical context. It could also indicate that influenza is only one of the possible pathogens causing an acute respiratory infection and that other pathogens, which we did not study, may play a role.

It is conceivable that influenza infections do occur more often in our control patients, since respiratory tract infections can cause a clinical picture that may be very similar to that of a pulmonary embolism. In fact, respiratory tract infection is one of the more common alternative diagnoses among patients suspected of having a pulmonary embolism [16].

Some aspects of the study require comment. First, for influenza detection we only used serum samples for the detection of viruses. Although the use of the complement binding assay makes false positive tests highly unlikely, for a more thorough detection, influenza genome detection in throat swabs or even nasal washes is preferred in combination with serum detection [17]. Furthermore, baseline serum samples have been collected at the time of admission to the hospital. If the influenza infection has occurred 1–2 weeks prior to admission, the antibodies may have already had peaked and a fourfold rise may not have been detected.

Secondly, the differences in patient characteristics between the two groups may have influenced the outcome. Since the effects of these differences are in opposite directions, we do not expect that the net effect has a major influence on the outcome of this study. Mean age in the patients with pulmonary embolism is slightly higher than in those without (58 vs. 53 years, respectively) and age is related with susceptibility for infection. The prevalence of asthma and COPD, which are also associated with susceptibility for influenza, was lower among the patients with pulmonary embolism. The degree of vaccination against influenza was not recorded in this study. Therefore we can not exclude whether there were less vaccinated subjects in the control group compared to the pulmonary embolism group, which could explain the difference in frequency of influenza infection. However, we expect that the degree of vaccination will be similar in both groups, based on the age distribution and numbers of subjects with a higher risk of influenza (as described in the results section). Therefore we conclude that vaccination against influenza probably does not significantly contribute to the differences in the frequency of infection with influenza. We adjusted the crude odds ratios for the following confounding factors: age, COPD and asthma. The adjusted odds ratios did not differ from the crude odds ratios (table 2).

**Table 2:** Association between influenza and pulmonary embolism.

	Odds ratios (OR) and 95% confidence intervals (95% CI)	
	
	Crude OR (95% CI)	Adjusted OR (95% CI)
Influenza A	0.22 (0.03–1.67)	0.22 (0.03–1.72)
Influenza-like illness	1.28 (0.75–2.18)	1.16 (0.67–2.01)

Odds ratios (OR) on the association between influenza A infection and influenza-like illness score and pulmonary embolism with corresponding 95% confidence intervals (95% CI). Crude OR are adjusted for age, COPD (= chronic obstructive pulmonary disease) and asthma. We did not calculate the odds ratio for influenza B, because there were only few infected patients (0 in the case group, 3 in the control group).

Since we compared our patients with pulmonary embolism to patients with symptoms of chest pain and/or dyspnoea, and not to a control group of randomly selected individuals, we still can not exclude the possibility that influenza leads to an increased risk of venous thromboembolism. However, the low rate of the very common influenza infection in the pulmonary embolism group, 1 out of 102 (1.0%), indicates that if this would be the case, this risk is probably only marginally increased. A large scaled study is needed to solve this issue.

We observed that the percentage of positive ILI scores was more or less the same in both patient groups, while the number of influenza infections was lower in the pulmonary embolism group. Influenza-like illness scores similar to our score are used in population based studies on respiratory tract infection and vaccination studies, in which the incidence of pulmonary embolism is low. Our results demonstrate that an influenza-like illness questionnaire, and thus symptoms of respiratory tract infection, can not be used to differentiate acute respiratory infection from pulmonary embolism in this clinical setting and has therefore limited utility.

In conclusion, this study suggests that influenza infection is not an important risk factor for pulmonary embolism. Symptoms of influenza are non-specific and can be present in patients with a pulmonary embolism. These symptoms can therefore not be used to differentiate between acute respiratory infection and pulmonary embolism in this clinical setting.

## Abbreviations

CFA = complement fixation assay; 95% CI = 95% confidence interval; COPD = chronic obstructive pulmonary disease; CVD = cardiovascular disease; ILI = influenza-like illness; SD = standard deviation; VTE = venous tromboembolism
